# Food security and nutritional status of children under-five in households affected by HIV and AIDS in Kiandutu informal settlement, Kiambu County, Kenya

**DOI:** 10.1186/s41043-016-0058-9

**Published:** 2016-07-22

**Authors:** Peter M. Chege, Zipporah W. Ndungu, Betty M. Gitonga

**Affiliations:** 1 Department of Food, Nutrition and Dietetics, Kenyatta University, P.O Box 43844-00100, Nairobi, Kenya; 2 Department of Nutrition and Dietetics, Jomo Kenyatta University of Agriculture and Technology, P.O Box 62000-01000, Thika, Kenya; 3 Department of Nutrition and Dietetics, Mount Kenya University, P.O Box 342-01000, Thika, Kenya

**Keywords:** Children under-five, Dietary practices, Food security, HIV and AIDS, Nutritional status

## Abstract

**Background:**

HIV and AIDS affect most the productive people, leading to reduced capacity to either produce food or generate income. Children under-fives are the most vulnerable group in the affected households. There exists minimal information on food security status and its effect on nutritional status of children under-fives in households affected by HIV and AIDS. The aim of this study was to assess food security and nutritional status of children under-five in households affected by HIV and AIDS in Kiandutu informal settlement, Kiambu County.

**Methods:**

A cross-sectional analytical design was used. A formula by Fisher was used to calculate the desired sample size of 286. Systematic random sampling was used to select the children from a list of identified households affected by HIV. A questionnaire was used to collect data. Focus group discussion (FGD) guides were used to collect qualitative data. Nutri-survey software was used for analysis of nutrient intake while ENA for SMART software for nutritional status. Data were analyzed using SPSS computer software for frequency and means. Qualitative data was coded and summarized to capture the emerging themes

**Results and discussion:**

Results show that HIV affected the occupation of people with majority being casual laborers (37.3 %), thus affecting the engagement in high income generating activities. Pearson correlation coefficient showed a significant relationship between dietary diversity score and energy intake (*r* = 0.54 *p* = 0.044) and intake of vitamin A, iron, and zinc (*p* < 0.05). A significant relationship was also noted on energy intake and nutritional status (*r* = 0.78 *p* = 0.038). Results from FGD noted that HIV status affected the occupation due to stigma and frequent episodes of illness. The main source of food was purchasing (52.7 %). With majority (54.1 %) of the households earning a monthly income less than US$ 65, and most of the income (25.7 %) being used for medication, there was food insecurity as indicated by a mean household dietary diversity score of 3.4 ± 0.2. This together with less number of meals per day (3.26 ± 0.07 SD) led to consumption of inadequate nutrients by 11.4, 73.9, 67.7, and 49.2 % for energy, vitamin A, iron, and zinc, respectively. This resulted to poor nutritional status noted by a prevalence of 9.9 % in wasting. Stunting and underweight was 17.5 and 5.5 %, respectively. Qualitative data shows that the stigma due to HIV affected the occupation and ability to earn income.

**Conclusions:**

The research recommends a food-based intervention program among the already malnourished children.

## Background

HIV is a global pandemic. Globally, 45 million people are living with human immunodeficiency virus (HIV) [1]. In Sub-Saharan Africa, about 22 million people are living with human immunodeficiency virus (PLHIV), while the number is about 1.3 million in Kenya [2]. The pandemic is having a significant impact on household food security as HIV and AIDS mainly strikes the most productive members [1, 3]. This in turn causes food insecurity in the affected household as the infected are not able to seek employment due to social stigma, which reduces working capacity and productivity [4, 5]. The family members also tend to devote more time in care giving to the sick members which would otherwise be spent in income generating activities. In addition, human immunodeficiency virus and acquired immune deficiency syndrome (HIV and AIDS) lead to increased use of resources, household income, and sale of assets to seek treatment [3, 6, 7]. Approximately 50 % of Kenyans live below the poverty line and live on less than $1 per day [8]. This situation is aggravated in households living with HIV [3].

The effect of HIV and AIDS on family structure and economic status has an impact on health and dietary practices [9, 10]. In most households, the quality of diet is compromised due to the low purchasing power [11, 12]. The effect of household food insecurity is greater on vulnerable populations like children under-five whose need for energy and nutrients are high due to rapid growth and development [13, 14]. Children from HIV-affected household are more vulnerable to food insecurity [15]. This is because they have increased reliance on external care due to the absence or sick condition of the parent or inadequate care from guardians who are mainly grandparents [16]. According to the National AIDS and Sexually Transmitted Infections Control Programme (NASCOP) [17], the largest populations of orphans in Kenya are from households affected by HIV and AIDS.

**Fig. 1:**
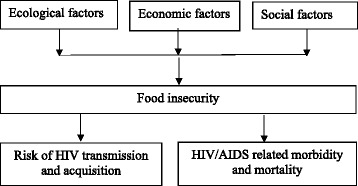
The predicting factors and the outcomes of HIV/AIDS; the various factors are ecological factors, economic factors, and social factors. HIV/AIDS results to a high risk of transmission, high case of morbidity and mortality

Informal settlements are associated with lack of adequate nutritious foods, inadequate clean water, and inadequate health care facilities. In addition, these areas are characterized by poor sanitation and poverty. Life is characterized by lack of infrastructure like housing, drainage, toilets, insufficient market supply, and extreme congestion [18]. This contributes to high prevalence of diseases and malnutrition in the slum settlements [19, 20]. The residents experience high levels of unemployment which affects their economic power [21, 22]. The predicting factors and the outcomes of HIV/AIDS are illustrated in Fig. 1.

In Kenya, the rate of under nutrition stands at 26, 4, and 11 % for stunting, wasting, and underweight, respectively [23]. This indicates that malnutrition is still a challenge among children under-five. According to Datta and Njuguna [24], enhancing food security is one of the interventions needed for households with HIV. The relationship between household food security and nutritional status among children from HIV-affected households in informal settlements is not well documented [25]. It is in this view that this study aims to assess food security and nutritional status of children 6–59 months from the affected households. This research focused on assessing household food security and nutritional status of children (6–59 months) in household affected by HIV and AIDS in Kiandutu informal settlement, Kiambu County.

## Methods

A cross-sectional analytical design was used to undertake the study. The target population was all the children under 5 years (6–59 months) from HIV- and AIDS-affected households in Kiandutu informal settlement. The bed-ridden children under-five and those on feeding programs were excluded from the study. A formula by Fisher was used to calculate the desired sample size of 260 which was increased by 10 % to cater for non-response [26]. Thus, 286 of children were included in the study. Purposive sampling method was used to select households affected by HIV and AIDS with children under-five. A list of all the households affected by HIV in the slum was generated through a census conducted by the community health workers, who are attached to the area. From the list, systematic random sampling was used to select the children from the identified households affected by HIV.

A researcher-administered structured questionnaire was used to collect data on socio-economic, dietary diversity, dietary practices, and anthropometry. Focus group discussion (FGD) guides were used to collect qualitative information on issues related to food security and nutritional status.

The questionnaire was pre-tested on 29 children while FGDs on 10 women. The pretesting sample was excluded in the final study sample. After the pre-testing, the tools were adjusted accordingly to ensure that all the data needed was collected. The respondents were the caregivers of the children under five in the affected households. The questions were translated to Kiswahili language. The weight and height of the child were measured using a bathroom scale and a height board, respectively.

Food security assessment was assessed using household dietary diversity score (HDDS) using 12 food groups (Swindale and Bilinsky [27]. Diet diversity score is a proxy indicator of quality of diets consumed by the household. The number of food groups eaten by household members in the previous 24 h was used [28]. A household with <4 food groups was classified as food insecure. Individual dietary diversity data for the child was collected separately.

A repeated 24-h recall was also used to determine the quality and quantity of the diet among the children where the amount of ingredient in each meal cooked as well as the volume cooked was recorded. The actual amount of food consumed by the child was also weighed. The amount of each ingredient consumed was then computed. A 7-day food frequency questionnaire was used to assess how frequently the various food groups were consumed within a week.

Three FGDs sessions each with 10 randomly selected caregivers were conducted after the quantitative data collection to generate more information.

### Statistics

Data analysis was done using statistical packages for social sciences (SPSS) (version 16.0). The quantitative data was summarized using descriptive statistics. The anthropometric data was transformed to nutrition indices (*z*-score values) by the use of the emergency nutrition assessment for standardized monitoring and assessment of relief and transition (ENA for SMART) software. Data collected from the 24-h recall was analyzed using Nutri-survey software for nutrient intake. Pearson Product Moment correlation coefficient was used to determine the relationships between dietary diversity score, dietary intake, and nutritional status. Qualitative data was coded and summarized to capture the emerging themes.

## Results

### Household characteristics

Data was collected in 274 households as 12 households did not respond or data was inconsistent. Data on household characteristics is shown in Table 1. From the study, fathers were the main household heads at 67.5 %. There were households headed by mothers (23.4 %) and grandmothers (6.2 %).

**Table 1:** Demographic characteristics of households

		*n*	%
Household head	Father	185	67.5
	Mother	64	23.4
	Grandmother	17	6.2
	Others	8	2.9
	Total	274	100
Parents living with HIV	Both father and mother	214	78.1
	Mothers only	48	17.5
	Fathers only	12	4.4
	Stepmother	3	1.1
Caregiver living with HIV	Mother	224	81.8
	Stepmother	17	6.2
	Grandparents	17	6.2
	Auntie	7	2.6
	Siblings	6	2.2
	Neighbor	3	1.1
	Total	274	100
Family structure	Both parent alive	160	58.4
	Only father alive	25	9.1
	Only mother alive	64	23.4
	Father and mother deceased	25	9.1
	Total	274	100

Most mothers inclusive of step mothers (31.5 %) were young between the ages of 26 and 30 years. The study noted that mothers established households as early as 17 years. A mother was quoted saying, “I dropped out of school at class five due to lack of school fees and got married.” From FGDs, this was attributed to the poverty in the slum area which leads to dropout from school, hence giving an opportunity for young people to engage early in family life. Among the households, 78.1 % had both father and mother living with HIV, 17.5 % were mothers. About 80 % of the parents were living with HIV or AIDS at the time of the study, and are therefore spending money on medication. About 20 % of these were unable to play the role of a caregiver.

The study noted that 65.6 % of fathers had attained primary education and above while for mothers, it was 55.6 % (Table 2). Some of the fathers (7.8 %) and 10.4 % of mothers had no formal education. Almost all of the grandmother caregivers had no formal education.

**Table 2:** Socio-economic characteristics of households

		*n*	%
Education of the father	None	14	7.8
	Primary incomplete	49	26.6
	Primary complete	83	44.9
	Secondary	29	15.6
	Tertiary	9	5.1
	Total	185	100
Occupation of the father	Business/petty trade	49	26.6
	Formal employment	14	7.6
	Casual laborer	92	49.7
	Farming	30	16.2
	Total	185	100
Education mother/caregiver	None	25	10.4
	Primary incomplete	84	34.9
	Primary complete	91	37.8
	Secondary	33	13.7
	Tertiary	10	4.1
	Total	241	100
Occupation mother/caregiver	Business/petty trade	52	21.6
	Formal employment	4	1.7
	Casual laborer	90	37.3
	Housewives	57	23.7
	Farming	38	15.8
	Total	241	100

Most fathers from the study (49.8 %) were casual laborers. For mothers, 37.3 % were casual laborers, 21.6 % engaged in petty trade, 23.7 % housewives, and 15.8 % engaged in farming activities. FGDs further highlighted that most of the caregivers reported that they experienced constraints in engaging to vigorous duties due to their HIV status and frequent episodes of illness as noted by a mother who highlighted that “When I am sick, my body is too weak to undertake my usual tasks”.

The mean monthly household income for the respondents was US$ 58.8 ± 4.1 with about 40.0 % households earning a monthly income of between US$ 46 and 65. From the FGD, the respondents indicated that the income was hardly enough to cater for their basic needs such as food, clothing, education, and medication. The study noted that more income (25.7 %) as given by a mean of US$ 15.1 ± 3.4 was allocated to medication as compared to 21.8 % allocated to food with a mean of US$ 12.8 ± 3.8. A mother noted, “I use most of my income to access medication than I use on food.”

From the study, majority of the households had four (29.3 %) or five (32.2 %) household members (Table 3). The average household size was 4.7 ± 0.12. Household size is a notable factor in food security and malnutrition.

**Table 3:** Household income and size

		*n*	%
Household income (US$)	<25	11	3.9
	26–45	28	10.2
	46–65	110	40.0
	66–85	96	35.1
	86–100	21	7.8
	>100	8	2.9
	Total	274	100
Household size	>8	11	3.9
	7	16	5.9
	6	32	11.7
	5	88	32.2
	4	80	29.3
	3	47	17.1
	Total	274	100

### Food sources

It was highlighted from the study that 63.9 % of the household purchased their food, and 27.4 % got their food from donations while 8.8 % produced the food they consumed in the household (Table 4). For those who produced, it was either in the kitchen garden, rented farm away from the slum or in the nearby swampy areas.

**Table 4:** Sources of food in households and dietary diversity score among children

		*n*	%
Sources of food	Purchase	175	63.9
	Donation	75	27.4
	Produce	24	8.8
	Total	274	100
Dietary diversity score	>4	65	23.7
	<4	209	76.3
	Total	274	100

### Food security

Most households (76.3 %) had a dietary diversity score of <4. The household dietary diversity score of 3.4 ± 0.2. This is evident from the high percentage of respondents who have to purchase food (63.9 %) amidst low incomes among the people living in informal settlements. Households that had low dietary diversity score were found to consume less number of meals consumed per day (*p* = 0.041). Household income had a significant relationship (*r* = 0.81; *p* = 0.039) where households with low income had low HDDS. The study shows individual dietary diversity of 4.1 ± 0.8 among the children.

### Number of meals consumed

The study noted that the number of meals consumed per day was (3.26 ± 0.07 SD). The number of meals consumed significantly (*p* < 0.05) related to the amount of nutrient intake namely vitamin A, iron, and zinc (Table 6).

### Food frequency consumption

From the food frequency questionnaire, the food groups that were frequently consumed by the children; more than four times in a week as per Food and Nutrition Technical Assistance (FANTA) guidelines [27], were leafy vegetables, milk, and cereals and at 91.2, 81.0, and 62.8 %, respectively (Table 5). Some of the food groups least consumed by the children in the study area were meats, fruits, and legumes. According to the information from FGDs, the frequency of food consumption was affected by the cost of food in the market and the level of household income.

**Table 5:** Proportion of children consuming >4 food groups

	*n*	%
Cereals/roots/tubers	222	81.0^a^
Milk	172	62.8^a^
Leafy vegetables	250	91.2^a^
Meats	58	21.2
Legumes/nuts	73	26.6
Eggs	28	10.2
Sugar	23	8.4

^a^Leafy vegetables, cereals, and milk were the most consumed foods

### Energy and nutrient intake

The mean energy intake was noted to be higher than the recommended daily allowance for children in each age category (Table 6). There was a significant relationship between the energy intake and nutritional status (*r* = 0.78 *p* = 0.038). Similarly, the intake of selected nutrients vitamin A, iron, and zinc intakes were also lower than the recommended by over 67, 61, and 43 %, respectively, of the children. Only 12.8 % had been given vitamin A supplementation.

**Table 6:** Mean energy and micronutrient intake as per age categories

Age in months		RDAs	Mean intake	% Taking adequate
6 to 11	Energy (Kcal)	1200	1080 ± 196	91.4
	Vitamin A (RE)	500	312 ± 52	28.6
	Iron (mg)	11	6.62 ± 0.01	37.1
	Zinc (mg)	3	2.6 ± 0.03	51.4
12 to 23	Energy (Kcal)	1200	1120 ± 182	90.8
	Vitamin A (RE)	300	312 ± 52	24.6
	Iron (mg)	8	6.62 ± 0.01	32.3
	Zinc (mg)	3	2.6 ± 0.03	50.8
24 to 35	Energy (Kcal)	1400	1260 ± 216	91.3
	Vitamin A (RE)	300	312 ± 52	26.1
	Iron (mg)	7	6.62 ± 0.01	34.8
	Zinc (mg)	3	2.6 ± 0.03	53.6
36 to 59	Energy (Kcal)	1400	1220 ± 209	88.6
	Vitamin A (RE)	400	312 ± 52	32.4
	Iron (mg)	10	6.62 ± 0.01	38.1
	Zinc (mg)	5	2.6 ± 0.03	56.2

### Nutritional status

The nutritional status of the children in this study was poor. The rate of wasting in this study was 9.9 % which was higher that national figures that stands at 7.0 % [23]. More children were found to be malnourished in ages 36–59 months than in other ages (Table 7). Stunting and underweight was 17.5 and 5.5 %, respectively.

**Table 7:** Nutritional status among the children as per age category

	Wasting			Stunting			Underweight		
Age in months		*n*	%		*n*	%		*n*	%
6 to 11	Severe	1	2.9	Severe	4	11.4	Severe	1	2.9
	Moderate	2	5.7	Moderate	11	31.4	Moderate	2	5.7
	Normal	32	91.4	Normal	20	57.1	Normal	32	91.4
	Total	35	100	Total	35	100	Total	35	100
12 to 23	Severe	1	1.5	Severe	3	4.6	Severe	2	3.1
	Moderate	4	6.2	Moderate	10	15.4	Moderate	4	6.2
	Normal	60	92.3	Normal	52	80.0	Normal	59	90.8
	Total	65	100	Total	65	100	Total	65	100
24 to 35	Severe	2	2.9	Severe	2	2.9	Severe	0	0.0
	Moderate	4	5.8	Moderate	10	14.5	Moderate	3	4.3
	Normal	63	91.3	Normal	57	82.6	Normal	66	95.7
	Total	69	100	Total	69	100	Total	69	100
36 to 59	Severe	4	3.8	Severe	2	1.9	Severe	0	0.0
	Moderate	9	8.6	Moderate	6	5.7	Moderate	3	2.9
	Normal	92	87.6	Normal	97	92.4	Normal	102	97.1
	Total	105	100	Total	105	100	Total	105	100

## Discussions

Energy and micronutrient intake correlated with both the number of meals and dietary diversity score (Table 8). It is recommended that children of this age consume at least three meals per day with snacks in between [28]. According to Gibson and Hotz [29], the more the number of meals consumed, the more the consumption of various nutrients.

**Table 8:** Relationship between number of meals and DDS kilocalories and micronutrient intake

		*r*	*p*
Number of meals	Kcal intake	0.426	0.021
	Vitamin A	0.478	0.029
	Iron	0.465	0.023
	Zinc	0.446	0.020
Dietary diversity score	Kilocalories intake	0.54	0.044
	Vitamin A	0.501	0.013
	Iron	0.514	0.023
	Zinc	0.514	0.020

Nutrient-dense foods are lacking in the slum. This explains why the mean intake of selected nutrients was below the recommended dietary allowance. The meals for children should be adequate, balanced, and should have diversity of nutrients to ensure proper growth and development as well as protection against diseases [30]. More children were wasted. According to Mittal et al*.* [31], nutritional status of children from poor resource center areas like slums is likely to be poor due to poverty.

The findings of this study are in agreement with studies which showed that the HIV and AIDS pandemic has increased the inability of affected households to put enough food on the table, possibly because of the continued decreased productivity in these households [3, 32]. Another study by de Waal and Tumushabe [12], confirmed that HIV and AIDS has such effects on the households as reduction in food quantity and quality as well as inability to afford foodstuffs that require cash inputs such as meat. This also agrees with findings from Masuku and Sithole [33], which revealed that the productivity of HIV-affected household members is reduced. This shows the need for support from a multi-sectoral approach in changing lives of people living in the informal settlement affected by HIV and AIDS.

In addition, the elderly have diseases associated with old age and reduced physical capacity to work [34]. According to a study by Mwawuda and Nyaoke [35], most household headed by females were found to have less income compared to male-headed households which is likely to impact on household food security. The children were grouped into age categories with majority (38.3 %) being in 36 to 59 months categories.

Engaging in early marriages could have contributed to the poor dietary practices adopted by the mothers. By leaving school to get married, the mothers are young and have minimal capacity to engage in income generating activities.

Education level is a determinant of the type of employment [2]*.* People with higher education are likely to be in better occupations. Better occupations have less physical strain. Qualitative data shows that the stigma due to HIV affected the occupation. The nature of occupation was reported to influence the household income. Inability to work translated to low income. This is in agreements with a study by Mwawuda and Nyaoke [35], which show that up to 45 % of PLHIV are unemployed. Most of the caregivers were mothers (81.8 %). Some children had grandparent, sibling, neighbors, and other relatives as caregivers who from focus group discussions were said to provide inadequate care to the children as compared to a mother. The number of children who were orphans was 41.6 %, have lost at least one parent. According to Kuo et al. [36], caregivers have a challenge of caring for children orphaned by HIV especially when they are also living with HIV.

According to the Government of Kenya National Aids and Control Council (GOK and NACC) [37], 50 % of Kenyans live below the poverty line and live on <$1 per day. Low economic power affects food security in both affordability and accessibility to nutritious foods. With most of the resources used to seek medication, the quality and quantity of food procured was affected.

Large household sizes have shown evidence of higher malnutrition than in small households due to sharing of available resources including food by many members [3, 14]. Food source is a determinant of food security especially if the main source is purchasing, and the incomes are low [24]. Household income affected food security in relation to ability to procure food. This is in agreement with the study by Gillespie [38], which found out that the household with more income was more food secure compared to those with low income.

## Conclusions

The socio-economic status in the study area was low. This is a main factor to food insecurity as the households have low incomes, which eventually affect the amount of food accessible to the household. High cost involved in management and treatment of opportunistic infections take a big share of household income. The inability of most affected people to seek employment due to social stigma and health issues reduces their ability to engage in activities to generate household income. HIV affects the engagement in income generating activities. Since most of the households depend on food procurement, food accessibility was affected. This resulted to food insecurity in the households leading to adoption of poor dietary practices. The lack of adequate food intake led to the poor nutritional status noted among the children.

The various coping mechanisms identified in the affected households contributed to the poor quality of life of all household members. In this current study in Kiandutu, the households adopted poor dietary practices which greatly impacted on the nutritional status of the children under five.

### Recommendations

This study recommends a food-based intervention program among the already malnourished children. Also recommended is a support to affected people through counseling so as to cope with social stigma in the society and place of work.

## Abbreviations

AIDS: acquired immune deficiency syndrome
ENA: Emergency Nutrition Assessment
FANTA: Food and Nutrition Technical Assistance
FGDs: focus group discussions
GOK: Government of Kenya
HDDS: household dietary diversity score
HIV: human immunodeficiency virus
NASCOP: National AIDS and Sexually Transmitted Infections Control Programme
NACC: National Aids and Control Council
PLHIV: people living with human immunodeficiency virus
SMART: Standardized Monitoring and Assessment of Relief and transition
SPSS: Statistical Packages for Social Sciences
